# Indigenous peoples and local communities as partners in the sequencing of global eukaryotic biodiversity

**DOI:** 10.1038/s44185-023-00013-7

**Published:** 2023-04-03

**Authors:** Ann. M. Mc Cartney, M. A. Head, K. S. Tsosie, B. Sterner, J. R. Glass, S. Paez, J. Geary, M. Hudson

**Affiliations:** 1 Genomics Institute, University of California, Santa Cruz, CA USA; 2 Te Kotahi Research Institute, University of Waikato, Hamilton, New Zealand; 3 Native BioData Consortium, Eagle Butte, SD USA; 4 School of Life Sciences, Arizona State University, Tempe, AZ USA; 5 Department of Fisheries, College of Fisheries and Ocean Sciences, University of Alaska Fairbanks, Fairbanks, AK USA; 6 Neurogenetics of Language, The Rockefeller University, New York, NY USA; 7 School for the Future of Innovation in Society, Arizona State University, Tempe, AZ USA

**Keywords:** Computational biology and bioinformatics, Environmental sciences, Scientific community, Eukaryote, Genome, Genomics, Taxonomy, Transcriptomics

## Abstract

The aim to sequence, catalog, and characterize the genomes of all of Earth’s eukaryotic biodiversity is the shared mission of many ongoing large-scale biodiversity genomics initiatives. Reference genomes of global flora and fauna have the potential to inform a broad range of major issues facing both biodiversity and humanity, such as the impact of climate change, the conservation of endangered species and ecosystems, public health crises, and the preservation and enhancement of ecosystem services. Biodiversity is dramatically declining: 28% of species being assessed by the IUCN are threatened with extinction, and recent reports suggest that a transformative change is needed to conserve and protect what remains. To provide a collective and global genomic response to the biodiversity crisis, many biodiversity genomics initiatives have come together, creating a network of networks under the Earth BioGenome Project. This network seeks to expedite the creation of an openly available, “public good” encyclopedia of high-quality eukaryotic reference genomes, in the hope that by advancing our basic understanding of nature, it can lead to the transformational scientific developments needed to conserve and protect global biodiversity. Key to completing this ambitious encyclopedia of reference genomes, is the ability to responsibly, ethically, legally, and equitably access and use samples from all of the eukaryotic species across the planet, including those that are under the custodianship of Indigenous Peoples and Local Communities. Here, the biodiversity genomics community is subject to the provisions codified in international, national, and local legislations and customary community norms, principles, and protocols. We propose a framework to support biodiversity genomic researchers, projects, and initiatives in building trustworthy and sustainable partnerships with communities, providing minimum recommendations on how to access, utilize, preserve, handle, share, analyze, and communicate samples, genomics data, and associated Traditional Knowledge obtained from, and in partnership with, Indigenous Peoples and Local Communities across the data-lifecycle.

## Introduction

According to the Intergovernmental Science-Policy Platform on Biodiversity and Ecosystem Services, “Indigenous Peoples and Local Communities (IPLC) are, typically, ethnic groups who are descended from and identify with the original inhabitants of a given region, in contrast to groups that have settled, occupied or colonized the area more recently”^1^. Approximately 37.9 million km^2^ of land and associated inland waters (spanning 82 countries) are owned or governed by Indigenous Peoples who represent <5% of the global population^2^. Indigenous lands traverse 25% of the Earth’s land surface, 40% of terrestrial protected areas and ecologically intact landscapes, and comprise 36% of intact forest landscapes^2,3^. Indigenous Peoples also act as a custodian for the species within these lands; for instance, 45–60% of threatened species in Australia are found on the lands of Indigenous Peoples^4^. It is also important to both note and promotes the contributions of IPLC women specifically to the transmission of associated Traditional Knowledge (aTK)^5^ involving genomics and to the sustainable use of biodiversity^6–8^. If also considering Local Community lands, these figures would most likely be greatly increased^9^. These statistics highlight the value and impact IPLC’ place-based knowledge, intergenerational practices, and intentional custodianship have on the conservation and sustainable use of global biodiversity and in-situ ecosystem health^10–13^. Many of these intergenerational systems precede those of westernized scientific approaches, including genomics^14–20^.

The plurality of knowledge systems and diversity of perspectives offered across IPLC is fundamental to achieving the mission to sequence all of life. There is, therefore, a need to prioritize IPLC participation and recognition across the biodiversity research enterprise and to build meaningful partnerships that are grounded in proactive, open, transparent, and accessible communication. Although open data is an important component of maximizing the scientific outcomes possible from biodiversity reference genomes, the fraught and ongoing history of extraction of biological resources and the aTK associated with genetic resources from the lands of IPLC cannot, and must not be ignored^21–23^. As the field of biodiversity genomics expands, mainstreaming a culture where IPLC’s authority to inform the stewardship of samples, data, and knowledge obtained within their jurisdiction on their own terms is fundamental^24^. In recognition of sovereignty, it is of equal importance to respect IPLC’s right to decline or defer participation in any research project if they so choose. In the words of Dr. Alex Brown, a Professor of Indigenous Genomics from the South Australian Health and Medical Research Institute, “We can only proceed at the speed of trust”.

**Fig. 1:**
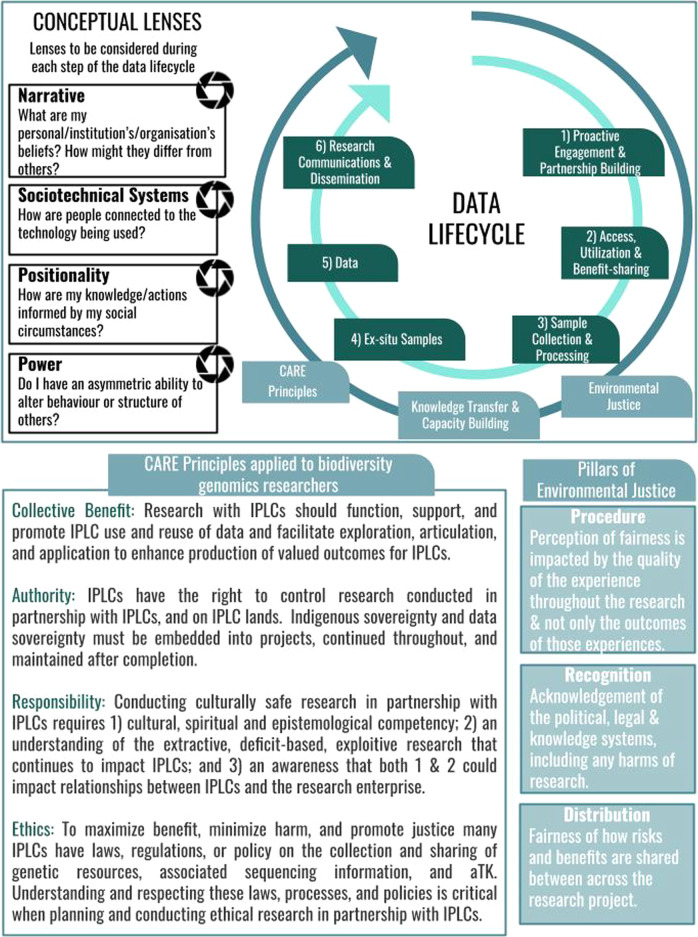
Reflecting on the data lifecycle through four conceptual lenses. Top right displays the data lifecycle, and the top left is the four conceptual lenses^33^ that researchers and research projects could utilize as tools to reflect upon throughout each step. The bottom right illustrates the three pillars of environmental justice, and the bottom left the CARE principles of Indigenous data governance, and how both are applicable across the data lifecycle.

**Fig. 2:**
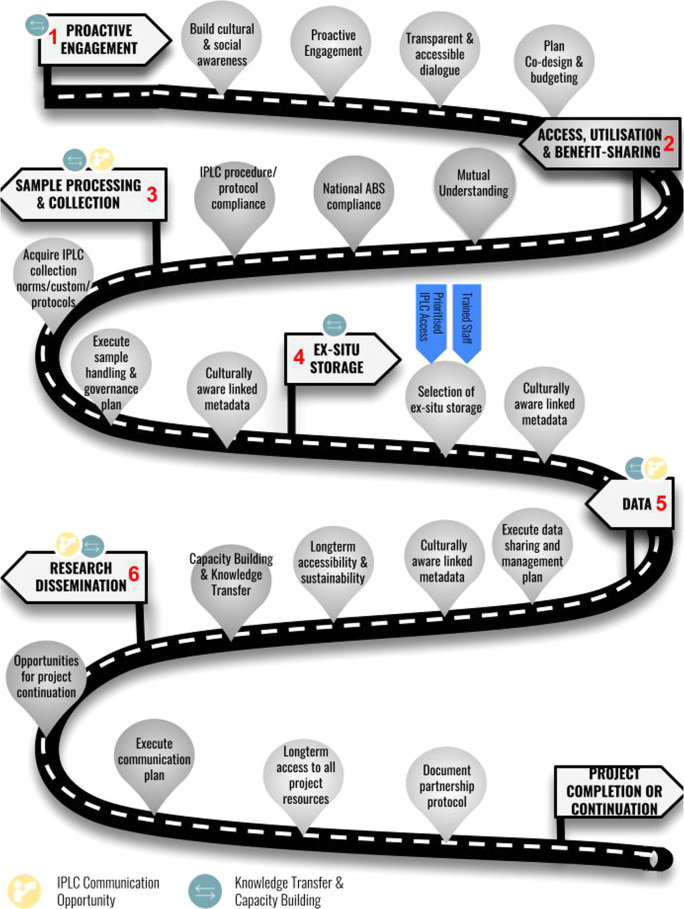
Framework roadmap. An overview of the proposed framework with report recommendations embedded, for building sustainable partnerships with indigenous peoples and local communities.

Here we offer a framework, grounded in environmental justice and the CARE principles (Collective Benefit, Authority to Control, Responsibility and Ethics)^25^, for biodiversity genomic researchers, projects, and initiatives to support and promote the building of trustworthy and sustainable partnerships with IPLC (Fig. 1). The CARE principles provide a set of broad principles for researchers to apply and implement when partnering with IPLC in research. However, they do not provide more granular, practical guidelines on how these principles ought to be applied in specific research fields. The intent of our framework is to support researchers in the field of biodiversity genomics to operationalize these principles across the data lifecycle from proactive engagement to communication and dissemination. By targeting the biodiversity genomics research community, we develop a tailored framework and provide: (1) context as to why the implementation of this framework is important for the field of biodiversity genomics; (2) granular, specific, and practical information on how to employ the framework through targeted recommendations; and (3) case studies to illustrate the recommendations detailed. We hope that this practical framework will expedite uptake across the biodiversity genomics community. We also provide minimum recommendations (Supplementary Section 1) on how to access, utilize, preserve, handle, share, analyze, and communicate samples, genomic data, and aTK obtained from and in partnership with IPLC across the data lifecycle (Figs. 1, 2).

The framework we present is based on a combination of lessons learned from empirical case studies and recognized best practices published in a wide array of relevant fields. Our recommendations represent an expert synthesis of issues, practices, and insights arising from previous efforts addressing data management international treaties and the CARE principles for genetic resources. All recommendations aim to put into practice existing local, national, and international legal instruments that promote the protection of Indigenous Peoples rights, including the Convention on Biological Diversity’s Nagoya Protocol^26^, United Nations Declaration of Indigenous Peoples (UNDRIP)^27^, United Nations Charter^28^, International covenant on economic, social and cultural rights^29^, the International covenant on civil and political rights^30^ and Vienna Declaration and Program of Action^31^. At the time of developing this framework, Indigenous Peoples have explicitly codified internationally accepted rights. It is important to note that Local Communities’ rights have, to date, not been codified in such a way, limiting their ability to participate in international fora and exercise their sovereignty in these spaces. Considering this, the recommendations provided within this manuscript align with the language of the CBD and Nagoya Protocol, which refer to Indigenous Peoples and Local Communities. The biodiversity genomic data lifecycle includes six steps: (1) Proactive engagement and partnership building, (2) Access, utilization, and benefit-sharing, (3) Sample collection and processing, (4) Ex situ storage, (5) Data, (6) Communication, and dissemination. Although most steps are considered sequential in nature, both steps 4 and 6 can happen in parallel to other steps. Our framework provides holistic guidance at each step of the data lifecycle on why and how to recognize IPLC rights and interests—supporting and promoting willing IPLC’ respectful, and systemic inclusion (Fig. 2). Whilst employing the framework, it is important to continuously reflect on the ethical practices and research processes being employed in order to keep human and societal concerns close to the technical aspects of the research projects, the day-to-day work of the researchers and overall project design decisions^32^. Figure 1 outlines a method for continuous ethical reflection using four conceptual lenses: narrative, socio-technological systems, positionality, and power^33^. Each step has a case study associated with highlighting a real-world example of the recommendations and framework (Supplementary Information Supplementary Section 2). Box 1 also provides a case study that highlights the implementation of the framework throughout each step of the project lifecycle.

Genomics is being mainstreamed as a tool to conserve and protect our biodiversity, but if it is to scale to create sequences for all of Earth’s biodiversity, it is important that we embed just, equitable, and inclusive practices into, and throughout, our biodiversity genomics research projects. Without commitment and intentionality, we risk defaulting to the normalized colonial practices that have and continue to exclude important segments of the population from the research enterprise, as well as the benefits generated. The framework we describe assumes that the biodiversity researcher, project, or initiative has already identified that a species of interest has a cultural, spiritual, or economical value to an IPLC; however, in many cases, researchers, projects, and initiatives may not be aware of this. To maximize the utility of this framework, prior to initiating projects, researchers should do their due diligence to identify whether the species to be accessed and used falls within the jurisdiction of an IPLC and if any aTK will be used. This way, IPLC have an opportunity to participate in the project and its design as outlined in the first step of the data lifecycle. We also acknowledge that for many Local Communities, there may not be a formal governance structure in place. In such cases, time should be taken to identify community partners who will be transparent about participation and accountable for decision-making and benefit-sharing within the Local Community. The research team should also acknowledge that, if there is no identifiable governance structure in place and there is no identifiable community partner with whom to engage in these conversations, then the research should not progress until these conversations have taken place with consent. Considerations for working in this space include, but are not limited to: (1) the research team is responsible for obtaining a deep understanding of the sociopolitical reality of the community and the norms and principles in place; (2) the engagement should be undertaken in a just, inclusive, transparent and accessible way to reflect the diversity of people and perspectives within the community; and (3) the consent mechanism should be transparent, clear, and appropriate for the community.

Box 1 **Framework implementation case study**. Conserving and restoring the endangered, culturally salient kākāpō through sustained partnerships The kākāpō, a flightless parrot endemic to New Zealand, was once common in the archipelago. Today, only 201 individuals remain. To inform the conservation and restoration of this species, genomic data were generated for the first chromosome-level reference genome, 35 modern genomes from the sole surviving island population, and 14 genomes from the extinct mainland population^97^. Here we outline the project lifecycle steps. An application for the study was submitted at the Kākāpo 125+ webpage detailing the names of researchers and collaborators, involvement of Māori researchers, benefit-sharing with Māori, and considerations of Mātauranga Māori. The submitted application was assessed and approved by DOC and Te Rūnanga o Ngāi Tahu. The project was then co-developed with Kākāpō125+, New Zealand Department of Conservation (DOC), Te Rūnanga o Ngāi Tahu, Genetic Rescue Foundation, Science Exchange, Experiment.com, Otago University, Duke University, Rockefeller University, and Genomics Aotearoa and funded by both private, public, and fundraising contributions. The kākāpo samples were obtained under an agreement of controlled access and management with respect to Indigenous data sovereignty, and that Te Rūnanga o Ngāi Tahu would maintain Kaitiakitanga i.e., governance and guardianship, over the data. Additionally, the study generated 13 genomes from ∼130-year-old museum specimens from extinct mainland populations^97^. The data remained the property of the New Zealand Department of Conservation, fulfilling its commitment to New Zealand Māori through the New Zealand Conservation Act 1987. The reference genome was agreed to follow the Vertebrate Genomes Project (VGP)^98^ standards, and all Kākāpō 125+ generated genomic data was generated following the assembly standards developed by Genomics Aotearoa. After this, re-sequencing of the historical specimens was performed by the Swedish National Genomics Infrastructure (NGI) at the Science for Life Laboratory and parallel sequencing and access to the UPPMAX computational infrastructure was supported by the Uppsala Multidisciplinary Centre for Advanced Computational Science. All genomes published by Kākāpō125+ have a README file detailing the creation, use, and statistics of each analysis. The reference genome assembly can be publicly accessed at the NCBI database under BioProject: PRJNA510145. The findings from this study are being used in kākāpō breeding and recovery led by the New Zealand Department of Conservation (DOC) and Te Rūnanga o Ngāi Tahu in the Kākāpō 125+ project.

### Step 1: Proactive engagement and partnership building

Openness and transparency are key for success when grounding partnerships between research projects and IPLC. Relational accountability, or holding yourself accountable to IPLC partners and their communities, is also a fundamental component to honorable partnership building and can be enacted through employing the four Rs of respect, reciprocity, responsibility, and relevance^34,35^. Whether it is a reference genome project using newly collected, previously collected, or revisiting archived resources obtained from IPLC or within IPLC jurisdiction, it is important to initiate discussions with IPLC in a manner consistent with customary laws, legislations, and protocols. Proactively reconciling differences in values, expectations, cultures, beliefs, knowledge systems, and worldviews that may exist between researchers and IPLC is critical for inclusive and equitable collaboration. An “ethical space” is defined as a safe place for different knowledge systems to interact with mutual respect, kindness, and generosity^36^. Establishing ethical spaces to explore how differences can co-exist with mutual respect as well as maintaining research integrity in these partnerships, requires careful planning.

The diversity across global IPLC requires the nature and extent of engagement to be tailored to the context and will depend upon the IPLC and project exploring a mutual partnership. It is recommended that researchers or projects seeking engagement first invest time into obtaining an understanding of the potential IPLC partner^6^. There are many publicly available resources to support a researcher’s journey toward cultural humility^7,37^, e.g., the e-learning modules provided by the NGO Natural Justice (https://naturaljustice.org/e-learning-modules/). A proactive review of the literature can establish whether the IPLC has existing guidance for research engagement (See Supplementary Table 2), but lessons can also be taken from guidance issued by other IPLCs or, indeed, the myriad of general guidelines and principles available e.g., Code of Ethics of the International Society of Ethnobiology^38^.

It is recommended that after a responsible level of competency has been built by the research team, and as early as possible and ideally prior to project initiation, IPLC are engaged through the appropriate designated IPLC representative(s) or organization. This is particularly important where cultural, linguistic, and social distance exists between potential IPLC partners and researchers, as the potential for miscommunication and misunderstanding becomes more significant. If engagement did not begin prior to project initiation, it is recommended that researchers seek to involve IPLC as soon as possible, revisiting and editing the research design when feasible.

A clear, transparent, and accessible dialog can facilitate a more balanced level of comfort, control, and power, but also establish trust from the outset. This dialog facilitates multiple evidence-based approaches to be employed during the project co-design stage^39^. Here, the goals of the project can be co-developed in a space where both scientific and Indigenous knowledge systems are treated as co-equal, with each system speaking for itself, without assigning a dominant knowledge system. After assessing complementarities and differences, the project can be co-designed to facilitate meeting the expectations of both the IPLC as rights holders and other stakeholders. It is important that the multiple evidence-based approaches is embedded in designing each step of the data lifecycle, so that the distribution of power across knowledge systems remains balanced (Supplementary Table 1) (Supplementary Information Case Study 1.1).

Co-design allows IPLC to determine what effect the project may have on their values and relationships but also provides researchers the opportunity to understand any IPLC practices, protocols, and processes and how best to embed them into the project. After co-design, it is important to take the necessary time to improve the “safety” of the project design in totality, considering the “Five Safes Framework”^40,41^. It may also be useful to co-develop more detailed plans for (1) sample handling and governance, (2) data sharing and management, (3) intellectual property and benefit-sharing, (4) communications, and (5) knowledge transfer and capacity building (Supplementary Table 1). If a partnership is mutually agreed upon, all agreements codified in appropriate consents can then be clearly and accurately bolstered into the project design so that every aspect of reference genome production is addressed and in alignment with IPLC expectations.

If, during a proactive engagement, an appropriate IPLC leader, entity, or organization cannot be identified, projects should not proceed to the next step of the data lifecycle as they will be unable to obtain the appropriate consent, potentially creating harmful impacts on the community members, and call into question the reputation of the project (Supplementary Case Study 2.1). Although researchers may need to wait to access and use samples in such cases, sustainable partnerships can still be built with partnering IPLC through capacity building and knowledge transfer.

### Step 2: Access, utilization, and benefit-sharing

IPLC are the custodians or rights holders of many of the most intact, sparsely populated, and biodiversity-rich locations across the world^2^. Therefore, establishing partnerships between genomic researchers and projects with IPLC, and ensuring IPLC rights are recognized, and that benefits are fairly and equitably distributed is of crucial importance to the success of biodiversity research. There is a fraught history of IPLC samples and aTK being unethically accessed, utilized, and claimed via the intellectual property (IP) (e.g., patenting and copyrighting) to misappropriate genetic resources without adequate consent or the equitable sharing of benefits^42–44^. Research carried out on genetic resources and the associated data can result in inventions that could be eligible for IP protection, and negotiating and granting access to genetic resources for research or commercial uses could also raise IP questions^44,45^.

Many international fora^46–49^, including the Convention on Biological Diversity’s Nagoya Protocol^26^, codify fair and equitable benefit-sharing from the access and use of genetic resources for the purposes of research and development when partnering with IPLC. Despite recognizing the sovereignty of Indigenous Peoples, the Nagoya Protocol is signed only by nation-states, and neither Indigenous Peoples nor Local communities are signatories. Therefore, it is the responsibility of each nation-state to both recognize and respect the sovereignty of all IPLC within their respective boundaries. Unfortunately, many nation-states fail to acknowledge or recognize self-identified Indigenous Peoples or Local communities, and so these communities are at risk of not benefiting from the Protocols provisions. To this end, it is of utmost importance that agreement methods that have been developed by IPLC, such as community protocols and customary law, for the access and utilization of genetic resources and aTK are considered and adhered to.

Community standards of best practices indicate that all genetic resources and aTK contributing to biodiversity projects must be both legally and ethically accessed and used in accordance with all existing applicable customary, local, regional, national, and sub-national laws, including those that govern the fair and equitable sharing of benefits. The CBD’s Nagoya Protocol entered into force in 2014 and provides provisions for a bilateral procedure between a user and a provider for access to genetic resources of potential value to be utilized for research and development purposes by the user consent and agreeing upon mutually agreed terms (MAT) with the provider’s Competent National Authority. The Protocol recognizes IPLC explicitly in Article 7. If a national procedure for Access and Benefit-Sharing (ABS) is in place, all researchers/projects are legally obligated to strictly adhere to these provisions. There are many publicly available resources to support research projects in determining their ABS obligations, including the CBD’s ABS Clearing House^50^ (See Supplementary Table 2). In cases where no national ABS procedures have been codified, IPLC may have alternative or additional practices, protocols, policies, and customary laws for access and use.

Typically, the first step of ABS procedures is ensuring a culturally appropriate level of consent is obtained. Consultation and full and effective participation of IPLC are crucial components of a consent or approval process. Free, prior, informed consent (FPIC) (Box 2) has become the preferred consenting strategy amongst many IPLC, and has been recognized by UNDRIP, CBD, and International Labor Organization Convention 169^51^.

A more in-depth exploration of the FPIC process has been developed by the Food and Agriculture Organization of the United Nations^52^. These guidelines provide detailed case studies to showcase successful FPIC procedures, but also offer an FPIC checklist to support projects seeking to establish a partnership with IPLC.

As documented in 2016, the Mo’otz kuxtal Voluntary Guidelines^53^ depending on the context of the partnering IPLC, FPIC, prior informed consent (PIC), or “approval and involvement” may be the appropriate form of consent to access and utilize samples obtained within the jurisdiction of an IPLC or obtaining any aTK. Consent approval refers to an agreement with the partnering IPLC or the competent authorities of those IPLC, as appropriate, to grant access to a potential user and includes the right not to grant consent or approval. Involvement refers to the full and effective participation of IPLC, in decision-making processes related to access to their aTK. If consent is successfully obtained, it is important that all appropriate measures are undertaken to ensure that samples and aTK are accessed and used in compliance to the terms of consent agreed upon.

If obtained, establishing a mutual understanding of the project is crucial prior to formally codifying MAT. A comprehensive MAT agreement addresses all aspects of the data lifecycle (Fig. 1) as well as sets forth terms under which benefits are to be shared, intellectual property is to be protected, and the mode of benefit-sharing. MAT also provides an opportunity to define the duration of the contract, the boundaries of the IPLC jurisdiction, and a process for conflict resolution. To date, IPLC have expressed an interest across a spectrum of monetary and non-monetary benefits, and a non-exhaustive list of potential benefits can be found in the Nagoya Protocol’s Annex^26^. Although much of biodiversity genomic research activity is wholly academic in nature and not aimed at the development of new products or processes, IPLC partners have the right to negotiate intellectual property (IP) rights on the research, development, and commercial use of IPLC genetic resources and aTK^54^ (Supplementary Information: Case Study 2.3). If IP protections are sought by the partnering IPLC, they can also be codified within MAT/material transfer agreements as contractual clauses. Model contractual clauses are available from the World Intellectual Property Organization’s Traditional Knowledge Division^55,56^. Researchers and research projects could benefit from seeking external expert advice on any relevant national legal systems in place, e.g., national patent laws, to better understand the contract review process and how contracts will be enforced. Notably, IPLC: (1) may seek agreements to ensure user access is conditional on not seeking IP rights and (2) may have additional privacy and confidentiality concerns. For example, an IPLC may stipulate a condition of access for non-disclosure of a certain aTK or may require that the specific origin of a rare, endangered, or culturally salient genetic resource be kept confidential. Further, for many Indigenous Peoples whose sovereignty remains unrecognized e.g., Indigenous nations in the U.S., many do not have the legal and research capacity to compete against law teams from major universities should there be a conflicting claim to IP^57^.

Finally, the ABS procedure is finalized through the issuance of an Internationally Recognized Certificate of Compliance (IRCC), which is uploaded to the ABS Clearing House by the provider country’s National Focal Point and Competent National Authority. Projects are then expected to take all measures necessary to ensure that IPLC samples and aTK are accessed and used in compliance with the IRCC.

Box 2 **Free, prior, and informed consent description** **Free:** Voluntary consent obtained, free from coercion, intimidation, and manipulation. The process should be designed and directed by the IPLC and unencumbered by external pressures or expectations. All members of the IPLC should be free to participate in decision-making processes.

**Prior:** Consent is obtained in the advancement of authorization and commencement of research activities. Consent requires the IPLC to be given time in advance to understand and assess key information about the research proposed to be conducted.

**Informed:** It is important that all information given to the IPLC is clear, consistent, transparent, comprehensive, and accessible (locally and culturally appropriate) and should provide a risk/benefit assessment of the proposed research. The personnel and location for information sharing should be culturally appropriate. Information should also be provided continuously throughout the consent process.

**Consent:** Consent should be collectively decided upon by the IPLC and reached through the decision-making processes determined by the IPLC. The granting or withholding of consent will be determined in accordance with the formal or informal political processes of each Peoples or community. IPLC may wish for consent to be regranted at specific project phases.

### Step 3: Sample collection and processing

aTK is dynamically evolving as IPLC ascertain new knowledge through further interaction with species. IPLC have garnered a wealth of wisdom on therapeutic applications, methods of use, harvesting, and cultivation of biodiversity. Further, aTK can inform abiotic environmental factors, ecosystem behavior and structure, species morphology, phenotype, taxonomy, growth, and much more^58–60^. Despite the importance of IPLC contribution to the conservation and sustainable use of global biodiversity, the access and utilization of IPLC samples and knowledge systems is not an entitlement. All contributions warrant fair recognition and respectful attribution, across the data lifecycle, including during sample collection and processing. Many standard fields address the legal expectations of sample collection and oversampling^61,62^ however, IPLC may have needs that go beyond these standards^63^.

As part of the initial engagement dialog, it is a good standard of practice to discuss aspects of sample handling and management and co-develop a plan (Supplementary Table 1). This plan can detail the species to be sampled, the cultural saliency or endangered nature of those species, and be used to help balance the risks associated with obtaining the required sample immediately against waiting for less invasive sampling techniques to be developed^64^. A plan is an important tool for recognizing IPLC input during species selection, and its co-development facilitates the recognition of western scientific and Indigenous knowledge systems. The plan could also include collection protocols to be followed, preservation, temporary storage, ex situ storage, sample handling during shipment, and return or destruction of samples after sequencing completion.

Upon species co-selection, species within IPLC jurisdiction should be collected and handled to obtain the freshest, best-preserved samples possible for DNA extraction to generate a reference genome that will meet accepted quality standards^62^. Careful consideration of the protocols utilized by the project when conducting field collection are pertinent to the success of species sample collection to avoid both sample wastage and the need for resampling. If ex situ storage of specimens is agreed upon in a codified agreement (Supplementary Section 2), it is important for projects to consider how best to sample, preserve and store additional tissues for both vouchering and cryopreservation. Noting that preservation requirements for long-read sequencing and proximity ligation sequencing may differ. This maximizes the scientific value of the sampling expedition, but also provides the IPLC the opportunity to explore further research questions they may have without the need to re-sample.

Metadata will be around far longer than the systems, organizations, and institutions that have generated it, and ensuring its interoperability with existing standards is the only way to safeguard its survival and scientific utility into the future. For all samples collected, generating robust metadata that is aligned with both the CARE^25^ (Fig. 1) and FAIR^65^ principles is fundamentally important. The FAIR principles have become a gold standard for genomics data management and sharing. They safeguard the integrity of data and maximize its scientific utility into the future by promoting the Findability, Accessibility, Interoperability, and Reusability of data. These principles center on responsible data handling, whilst the CARE principles provide support for centering Indigenous values during data handling. Many have identified potential incompatibilities between the FAIR and CARE principles^66^, specifically related to open data sharing. However, the CARE principles were developed to act in complement with the FAIR principles to ensure that Indigenous data remain FAIR whilst centering Indigenous sovereignty. Innovative metadata tools have been developed to harmonize the FAIR and CARE principles for genomics data in open digital environments e.g., the Local Contexts Labels and Notices system.

It is critical to consider what, how, and where metadata ought to be collected, accessed, and stored and for this to be mutually agreed upon with the partnering IPLC (Supplementary Sections 1, 2). As previously mentioned, interoperability is crucial to streamlining downstream connections to vouchers in museum collections, biobanks, and digital data, and where appropriate, metadata should align with pre-existing standards such as Darwin^67^ and Dublin Core^68^. To solidify Indigenous Data Sovereignty^69^ robust information on (1) provenance about place, people, and processes, (2) access and use permissions of samples, and (3) community protocols for use and reuse is important. Additionally, recent studies have also highlighted the importance of clearly documenting the cultural importance of species (CIS) to establish and retain the link between people and nature within metadata^70^. However, it is worth noting that respecting IPLC rights and interests may require standard metadata collection processes and standards to be adapted and refined to respond to the needs, wants, and wishes of the partnering IPLC. In some cases, metadata could be identified as culturally sensitive by the IPLC, so if projects redact metadata, this information can be mapped to the *dwc:informationWithheld* field e.g., culturally important sites. Moreover, metadata requiring generalization can be mapped to *dwc:dataGeneralization* e.g., geographical coordinates of an endangered species.

One process under development for the inclusion of IPLC rights into metadata is the TK (Traditional Knowledge) and BC (Biocultural) Labels and Notices^71^, developed by Local Contexts^72^. The TK and BC Labels and Notices are an extra-legal digital intervention addressing issues of provenance, ownership, access, control, and governance over IPLC digital collections and data. Addressing Indigenous interests that sit outside the current IP regime, the TK and BC Labels and Notices were initiated to directly bring Indigenous authority, perspectives, and protocols into the digital management of IPLC collections in museums, libraries, and digital environments. The TK and BC Labels and Notices provide a much-needed template to address the recognition of the inherent authority and responsibility of IPLC over the genetic resources of flora and fauna, as well as all associated sequence information and aTK. The Labels also facilitate the attribution of samples, data, or aTK across multiple communities using the TK MC Label. Importantly, both Labels and Notices can be associated with both genetic resources and associated sequencing information, as they have a permanent, unique ID that can be entered into the metadata. Supplementary Table 3 highlights how the Labels and Notices can be entered in order to remain interoperable with pre-existing metadata schema.

If multiple communities have aTK associated with the same species, each IPLC can register this through the Local Context Hub and issue a TK MC (multiple community) Label. The Label can be customized to disclose specific information concerning how to access, utilize and share benefits from the aTK. It can be challenging to understand the often-complex relationships that IPLC have with their territories and each other. Interests can overlap, or the distribution of a particular species might cross the territories of many different communities. It is better to surface than silence this complexity and allow IPLC’s to determine how their respective interests should be recognized. Sites like Native Lands (www.native-land.ca) can be useful resources to identify communities with associations to the territory or land where the samples were collected, and the TK MC Labels provide a transparent platform to support dialogs. The Labels also facilitate potential future repatriation/rematriation efforts of the samples, aTK, and sequencing information in accordance with the CBD-adopted “*Rutzolijirisaxik Voluntary Guidelines”*^73^ on ethical repatriation/rematriation of IPLC samples.

### Step 4: Ex situ samples: taxonomy, vouchering, and biobanking

When ex situ samples are collected, preserved, and stored with the effective participation of IPLC, they can be of great benefit to the IPLC and the biodiversity scientific enterprise at large. There is an emerging expectation that IPLC should be actively involved in the governance of data; however, this will be subject to national legislation and community capacity. The value of natural history collections only continues to increase as digitized vouchers become more common. Vouchering has many scientific benefits, such as enabling verification of species identifications, and understanding historical changes in climate, pollutants, and disease, but also enables improved governance. For example, well-documented voucher specimens make it possible to verify a specimen’s provenance and provide opportunities for outside entities to evaluate collection and dissemination practices (e.g., journal editors and funding agencies)^74^. Other options for ex situ sample storage include the use of biobanks. Biobanking samples facilitate reuse for further research, results verification, and limits the collection and sacrifice of another individual from the same species. Again, cryopreserved samples can be of great benefit to IPLC as this method can reduce the need for resampling and allow the IPLC to conduct research into the future without sacrificing another species. However, power asymmetries exist due to the lack of museum collections and biobanking facilities within IPLC jurisdictions. Additionally, there is a fraught history of biodiversity samples and knowledge being extracted from Indigenous lands, that roots back to the beginning of colonization^75^. These problematic practices have led to many Indigenous samples and knowledge being stored, accessed, and utilized by those outside of the country/territory of origin. The legacy of these practices can still be seen in institutional collections today^76,77^. Many of these extracted Indigenous resources have since been used for research outputs (“helicopter research”)^78–80^ or have been commodified (“biopiracy”)^81–83^ with no benefits being shared back to the community. A transformative shift away from these colonial practices is needed where all terms for the access and use of vouchers and biobanking purposes are included, mutually understood, and agreed upon in the consent and codified within contractual agreements (Supplementary Section 2).

Ideally, IPLC samples would be stored, shared, and managed by the partnering IPLC within its jurisdiction. However, the significant initial and continuous investment required to establish ex situ permanent collections and biobanks has resulted in an uneven global distribution^84^, and so the partnering IPLC may wish to pursue the long-term storage of samples outside of IPLC jurisdiction. For IPLC samples housed outside of IPLC jurisdiction, several factors are helpful to consider. Ideally, an ex situ storage strategy would be co-developed and agreed upon in the sample handling and management plan (Supplementary Table 1) during the initial proactive dialog (Supplementary Section 1).

Reflecting on the ongoing and historical harms caused to IPLC due to inappropriate and exploitative ex situ IPLC sample collection, storage, and preservation practices^85^ can help to frame and understand any rightful distrust or unwillingness IPLC may have in depositing samples into an ex situ entity. The diversity of IPLC attitudes toward ex situ sample collection reflects the unique history, culture, and values of each distinct IPLC^86^. After building an appropriate level of cultural awareness, ex situ collection can then be both discussed during the proactive engagement (Supplementary Section 1), consent obtained, and terms of access, use, and benefit-sharing codified (Supplementary Section 2).

Co-selecting an appropriate ex situ entity can (1) support the respective IPLC culture and values, (2) provide rightful attribution to the IPLC, (3) store culturally salient metadata consistent with Dublin Core and Darwin Core standards (Supplementary Section 3), (4) provide cultural protocols to guide the stewardship of IPLC samples within the entity, and (5) document and work toward the coexistence of western with traditional names for organisms to facilitate their propagation through to digital databases (Supplementary Information Case Study 3.1)^87,88^. Some projects also consider subdividing samples across multiple institutions in case of a catastrophic event at one and potential loss of the sample. Notably, alternative, more comfortable entities may exist that can act as a “safe harbor” for IPLC samples offering additional protections not possible from a typical institution within a non-IPLC jurisdiction (Supplementary Information Case Study 3.2). Another helpful approach could be for projects to explore options to hold IPLC samples in trust until the partnering IPLC wishes to reclaim them.

The CBD-adopted “*Rutzolijirisaxik Voluntary Guidelines*” encourages researchers to identify samples unethically or illegally collected from IPLC lands and consider repatriating/rematriating these samples to the relevant IPLC in recognition and respect of IPLC sovereignty. Natural History Museums are increasingly confronting the problematic basis of many of their collection items^75^, including a request to the Natural History Museum in London for the return of a 12,000-year-old giant ground sloth specimen collected without permission in 1890 (*Mylodon darwinii*) to Chile and the successful repatriation/rematriation of *Pelagornis chilensis* from Senckenberg Museum to the National Museum of Natural History in Santiago^89^. The Indigenous Research Protection Act and Indigenous research guidelines in Canada, New Zealand, Australia, and the United States all address provisions for individual or collective withdrawal of genomics samples^90^.

### Step 5: Data generation and handling

Fundamental to the success of generating reference genomes for all of Earth’s biodiversity is ensuring responsible and safe data management, sharing, and analyses that support and respect the needs and rights of partnering IPLC^41^. To date, the benefits of research produced by data obtained from IPLC lands, or in partnership with IPLC, have not met the needs of, or have been inaccessible to IPLC—perpetuating structural injustices. Addressing structural injustices remains a challenge due to their systemic and pervasive nature including a lack of IPLC governance and authority; power imbalances; unresponsive research; and insufficient inclusion of IPLC as partners in the biodiversity research enterprise. Mitigating injustices requires investment in genomic infrastructure, building IPLC capacity to enable sovereignty, and safeguarding IPLC stewardship of the data associated with IPLC resources.

Kukutai & Taylor define data sovereignty as the right of Indigenous peoples to determine the means of collection, access, analysis, interpretation, management, dissemination, and reuse of data pertaining to Indigenous people from whom it has been derived, or to whom it relates. Indigenous aspirations for Indigenous Data Sovereignty are being articulated as a response to being excluded from participation in research and any intellectual property generated from those activities. Greater awareness of the ways in which IPLC’ can be marginalized from opportunities across the data lifecycle have contributed to the growing advocacy for greater involvement throughout the process, including recognition of pre-existing rights and those affirmed through UNDRIP and other instruments. Ideally, both sample preparation and subsequent sequencing will take place within the jurisdiction of the partnering IPLC. IPLC may also wish for data to be specified, the internal or external, data repository (Supplementary Information Case Study 4.1). However, this may be infeasible given inequitable access to the technology and scientific equipment required for generating high-quality genomic datasets. In such cases, equitable, collaborative, and inclusive partnerships that involve and consult IPLC across the data lifecycle are paramount to safeguarding the return of both short and long-term benefits to the IPLC (Report Supplementary Sections 1–3). Topics for consideration during the proactive engagement (Supplementary Section 1) whilst co-developing a data handling and management plan (Supplementary Table 1) are outlined below.

First, it is important that all cultural considerations stated in the sample handling and management plan (Supplementary Section 1 and Supplementary Section 3) are considered during sample transportation and handling when shipping and handling samples outside of IPLC jurisdiction. Detailing a process for culturally appropriate destruction or the return of specimens post-sequencing within this plan can be helpful as the sample destruction upon sequencing completion may be culturally damaging, and the return of such specimens to IPLC jurisdiction could be preferred.

All sequencing data generated should be associated with culturally relevant, interoperable, metadata to safeguard fair attribution and encourage and promote benefit-sharing (Supplementary Section 2). Including robust provenance, permission, and protocol metadata information is important and should be consistent with both Dublin Core and Darwin Core standards, but also linked to the original metadata collected upon sample collection (Supplementary Section 3 and Supplementary Table 1). As mentioned in Supplementary Section 3, data infrastructures are increasingly interoperable and standardized, so it is vital that space is created for Indigenous metadata within an existing schema. The TK & BC Labels and Notices, outlined previously, can also be used for genomics sequencing information metadata and maintain such interoperability (Supplementary Table 3).

IPLC may have expectations, such as the CARE principles (See Box 3), for how their data may be accessed, stored (short and long-term), used, and reused. Proactively documenting these preferences in the data handling and management plan provides clarity to downstream users. Indigenous Data Sovereignty is essential if selecting an external repository, and thoughtful consideration can ensure that the selected repository supports the appropriate, culturally tailored data governance for access and use of data as dictated by the IPLC. In cases where it is agreed that IPLC data are housed in an external data repository, a repository that endorses and implements the TRUST principles is highly recommended^91^. The TRUST principles include the principle of sustainability, which importantly safeguards the long-term data storage of IPLC data so that IPLC can sustainably gain access beyond project completion. Prioritizing an accessible format for IPLC data that is useful for the needs of the IPLC promotes the reusability of the data for the IPLC.

Embedding analyses that support research questions of importance to the partnering IPLC into the project design safeguards beneficial research outcomes returning to the partnering IPLC. Additionally, IPLC participation in the data analysis process is important for equity and inclusion; this may require funding to support IPLC partners’ travel to collaborating institutions to access analysis resources as well as time and resources to train IPLC in downstream analysis in their own jurisdictions.

As equal partners, ensuring IPLC are the data stewards over genomic data generated from species within IPLC jurisdiction is important for building capacity building, but also IPLC participation in the digital revolution. Prioritizing opportunities to build capacity, leveraging IPLC existing capacities (asset-based approach^92^), and ensuring alignment with IPLC needs, objectives and motivations can facilitate, strengthen, and expedite IPLC stewardship. This could include training, workshops, mentorship in laboratory techniques (DNA extraction, library preparation, etc.), and data analysis techniques (QC, assembly, downstream analysis, etc.). This may also include subsidized access to infrastructure (e.g., high-performance computer cluster access) and funding equipment and infrastructure (e.g., writing IPLC partners in grants to purchase supplies, equipment, and computational resources). Sequencing data is more often used locally where the samples have been originally collected^93^, so it is important to intentionally build local capacity for the full scientific value to be realized. This can also help to ensure that IPLC are supported in removing barriers to implementing the research findings. When constructing capacity-building opportunities, socioeconomic factors must be considered so that equity and accessibility to all members within the IPLC can be achieved^6^.

Box 3 **Indigenous data governance**. Application of CARE principles to biodiversity genomics data governance **Collective Benefit:** Ensuring data management and sharing functions, supports, and promotes IPLC to use and reuse IPLC data facilitates IPLC exploration, articulation, and application and enhances the production of outcomes of value to IPLC.

**Authority:** Indigenous Data Sovereignty^99^ affirms IPLC authority to control how IPLC data is expected to be used/reused, analyzed, interpreted, managed, and shared. It safeguards IPLC governance, and data held by non-IPLC entities actively involves IPLC in stewardship decisions.

**Responsibility:** Conducting culturally safe biodiversity research through appropriate sharing and use of IPLC data were the responsibility of researchers. Awareness and recognition of the historical and ongoing research misconduct involving IPLC and IPLC data are important for understanding, and respecting IPLC legitimate research-related concerns. Proactive, transparent conversations around how IPLC data will be managed and shared are of paramount importance to building trust and cultivating genuine relationships between IPLC and the biodiversity genomics research community.

**Ethics:** To maximize benefit, minimize harm, and promote justice, many IPLC have laws, regulations, or policy on the management and sharing of sequencing information and aTK acquired from IPLC lands. Recognizing these laws, processes, and policies is critical when planning and conducting ethical research that involves generating, managing, and sharing IPLC data.

### Step 6: Research communication and dissemination

Peer-reviewed publications are the primary research communication strategy utilized by academic researchers to communicate research findings. These publications serve to promote and consolidate the associated researchers’ academic career, and unfortunately, they are often prioritized by researchers over aims relevant to other project stakeholders and partners. The heterogeneity across global IPLC engenders diverse expectations surrounding the communication and dissemination of results, contribution acknowledgements, roles and responsibilities, and timeframes. Honoring a process of openness and transparency in developing partnerships between biodiversity genomics research projects and IPLC must include thoughtful and intentional participation to (1) effectively report research findings back to the partnering IPLC throughout the research project, (2) communicate research findings to stakeholders both inside and outside of academia, and (3) ensure benefits are shared related to dissemination and communication from research engagement.

Developing a research communication and dissemination plan as part of a transparent process during partnership building with IPLC is a critical step to proactively address prior to project initiation (Supplementary Section 1, Supplementary Table 1, and Supplementary Information Case Study 5.1). Projects can use the plan to ensure a strategy is in place to implement its contents, noting that as the dissemination and communication cycle is iterative in nature, a process for quality control and improvement throughout the lifecycle of the project may be needed. Careful planning can ensure that all party’s values, relationships, and procedures are understood and clearly delineated for informal (e.g., Twitter, workshops, trainings, news, and other media, etc.) and formal (e.g., academic journals, press releases, theses and dissertations, etc.) research communication and dissemination. Critical to success is including how the project can best acknowledge IPLC participation in all research outputs. Research translation (i.e., transforming scientific evidence for use in practice) involves many practices and strategies and operates at varying levels, including the individual, project, institution, community, and society.

The plan is also useful for defining the dissemination objectives, prioritize target audiences or stakeholders, identify potential key messages, and detailing other work plans (e.g., timelines, responsibilities, budgets, and communication channels such as press releases or journals). IPLC may have pre-existing and preferred platforms or modes of communication platforms in place; identifying and prioritizing these primary platforms of communication and dissemination during plan development is recommended. Additionally, on project completion, IPLC may request a final research report from the project. This allows the research findings to be communicated back to the partnering IPLC but also provides a means for outcomes to be transmitted back to all who participated in the research project, including IPLC leaders or designated officials, IPLC entities or organizations, IPLC members, informal networks, and colleagues. This report could be written or oral in format and may require plain language summaries or translation to ensure that results are as accessible as possible to the IPLC. Translation to local languages is important for both academic goodwill, but also so that the report is useful for local policymakers. Free, online translation tools such as DeepL or Google Translate can be useful when a paid translator has not been accounted for in the project’s budget. Other culturally accepted forms of communication, e.g., ceremony, might also be avenues for a final research report.

After the mode of communication and expected timeline for sharing research outcomes have been mutually understood and agreed upon, it is important to establish a process for IPLC feedback on the effectiveness of the research study and its findings. In alignment with the Tri-Council Policy Statement^94^, it is a recommended best practice to recognize and integrate feedback and suggestions from the partnering IPLC in any publication associated with the research project, as this can strengthen research findings with additional complementary information and prevent the publication of misunderstood or misrepresented findings. If an unresolvable disagreement concerning the interpretation of the research findings arises, options include either (a) providing an opportunity for the IPLC to make its views known or (b) accurately reporting any disagreement about the interpretation of the data in all research communications. This method facilitates the publication of the research findings whilst respecting IPLC knowledge systems as co-equals when interpreting and contextualizing the findings.

## Conclusions

Our framework intends to support biodiversity genomics researchers, projects, and initiatives in making an intentional commitment to the governance, knowledge systems, sovereignties, and self-determination of current and future generations of IPLC. We acknowledge that the applicability of each recommendation may vary depending on the characteristics and aspirations of the IPLC. However, we strongly believe that engaging in our proposed framework will help build bridges between IPLC and the genomic science enterprise and boost the reputation of the biodiversity genomics research process. Progress in this space will only benefit all who are engaged in the pursuit of the conservation and sustainable use of global biodiversity. We hope this framework will be revised and revisited iteratively to include more IPLC perspectives and voices.

We are cognizant that executing this framework and building sustained partnerships can often require a significant investment of time and resources from both IPLC and research projects. However, we believe it can achieve long-term relationships with IPLC that support the conservation and management of many culturally salient, endangered, and endemic species under their custodianship. To maximize the effectiveness of these sustained partnerships, it is important to document the protocol. This can provide useful insights to other projects on the procedure and can also be useful to the partnering IPLC for re-utilization in future research partnerships—alleviating the burden. A documented protocol could also benefit both the partnering IPLC and the project if multiple species are sequenced from the outset.

In some cases, multiple communities may have an aTK associated with the same species, and the interests of these communities may not always align. In such cases, we encourage researchers to make an effort to disclose their association (TK MC Label) and facilitate an opportunity for a dialog between the communities, if appropriate. IPLC may have pre-existing, non-Western methods for resolving such conflicts, which should be respected. Resolving disputes in advance is important prior to project initiation and continuation.

Building sustained partnerships with IPLC in the current funding setting can be difficult, and in order to be truly successful must have a dedicated budget. Our framework outlines the importance of proactive dialog and co-design. Ideally, projects would conduct these activities prior to grant submission. Moreover, engaging with an IPLC requires time and resources from the community. The knowledge, time, and investment before project initiation deserve fair compensation. Unfortunately, many grants do not reimburse projects for these proactive activities that take place before submission.

Additionally, funding bodies’ open data-sharing policies may risk IPLC willingness to participate. Mandating openness can inhibit an IPLC ability to exercise Indigenous Data Sovereignty^95^. More inclusive data-sharing policies will be vital to including IPLC in future biodiversity genomics research.

The worldwide biodiversity genomic community’s mission to mainstream the utilization of a reference genome as a tool to understand, protect, and conserve global diversity cannot be achieved without the recognition, participation, and equitable distribution of benefits to the custodians of the majority of our remaining biodiversity, Indigenous Peoples and Local Communities. The biodiversity crisis has been predominantly caused by anthropogenic drivers, particularly land-use change and over-exploitation. However, the global human population has not contributed to this crisis equally, and those who have contributed the least, IPLC and the Global South, stand to be impacted the most (e.g., 33% of biodiversity impacts in Central/South America and 26% in Africa are driven by consumption in other regions^96^). As the biodiversity genomics community grows, it is critical to ground project ambitions to sequence all life in an awareness of the sociopolitical realities, the plurality of knowledge systems, and non-genomic tools that together can achieve the greater mission of protecting and conserving the world’s biodiversity.

Finally, by mainstreaming our framework across the field of biodiversity genomics, we hope it will move beyond simply a set of best practices and toward a community-adopted standard of practice, where researchers acknowledge the value of implementing the framework and are inspired to innovate tools for monitoring success and compliance across the community.

## Supplementary information

Supplementary information
