# Supplementary material for: Indigenous peoples and local communities as partners in the sequencing of global eukaryotic biodiversity
Source: NPJ Biodivers. 2023 Apr 3;2:8. doi: 10.1038/s44185-023-00013-7 (PMC11062294; doi:10.1038/s44185-023-00013-7)
Supplement: Supplementary file 1 — Supplementary information [file 44185_2023_13_MOESM1_ESM.pdf]

Indigenous Peoples and Local Communities as partners in the sequencing of global eukaryotic biodiversity

Mc Cartney, A.M*., Head, M.A.,Tsosie, K.S.., Sterner, B., Glass, J.R.., Paez, S., Geary, J., Hudson, M

Supplementary Section 1: Recommendations

Step 1 Proactive Engagement and Partnership Building

- Investing the time and resources necessary to develop genuine relationships with IPLC(s) is necessary to gain support for the project, obtain the necessary permit(s) or equivalent, and establish mutually agreed terms for sample access and utilization. Building cultural and social awareness of the IPLC(s), including any societal or gender roles, across the project is a key first step for trust and relationship building.
- In cases where a research project proposes to sequence a species from a sample accessed within IPLC jurisdiction, or where aTK is to be used, a proactive engagement with the IPLC(s) helps to ensure IPLC(s) participation in defining the project's aims and design. This also provides an opportunity for IPLCs to suggest additional or alternative species of pertinent interest.
- To more fairly distribute power during an engagement with IPLCs, a transparent and accessible description of all aspects of the proposed project provides an opportunity for IPLC(s) to contribute meaningful input and direction on each aspect.
- Co-develop plans to steer the direction of the project from initiation to completion, noting the importance of gender balance in decision making processes. Useful plans to consider include Sample Handling and Management, Data Handling and Management, Intellectual Property, Benefit-sharing, and Communication.
- In cases where an IPLC sample or aTK has already been accessed and utilized but insufficient information is available about the legality of access and utilization there may be requirements to be followed under national legislation. If no legal requirements exist. an engagement with the IPLC can help to obtain an access permit or its equivalent and establish mutually agreed terms, or indeed inform whether it would be best for utilization to be discontinued.

Step 2 Access, Utilization and Benefit-sharing

- Compliance with any national Access and Benefit -Sharing procedures in place where species samples, or aTK, are being accessed is a legal obligation. For the Nagoya Protocol, Access and Benefit Sharing procedures typically codify a protocol for both consent (Prior Informed Consent or preferably Free Prior Informed Consent) and mutual agreements (Mutually Agreed Terms) between a provider IPLC and a recipient (researcher/project).
- In cases where no national Access and Benefit-Sharing procedure exists, IPLCs may have alternative, culturally appropriate methods e.g., community protocols and customary law for formalizing agreements as dictated by an appropriate designated IPLC entity, organization, institution or leader.
- Mutually agreed terms can detail terms for sample access and use (re-use), third party transfers, intellectual property rights, contract duration, conflict resolution and monetary/non-monetary benefits to be shared. To ensure ethical and legal compliance, a mutual understanding of the terms within this contract is important.
- If agreed that samples can be transferred to a third party this can be explicitly stated within the mutually agreed terms and can promote trust and transparency. As a best practice prior to transfer, providing documentation to the recipient entailing the purpose of utilization, period of license, benefit-sharing (if stated) expectations, and additional terms and conditions of use prior to transfer can help ensure that the recipient's intended use is in alignment with the IPLCs expected use.

Step 3 Sample Collection and Processing

- Prior to collection, an understanding of any IPLC cultural sensitivities, customary laws, norms and protocols surrounding the collection, destruction, long-term storage or return of IPLC samples after project completion can help to culturally tailor a sample handling and governance plan design.
- The continuous involvement of an appropriate designated IPLC entity or leader throughout the collection process can be helpful in supporting the project to ensure ethical and sustainable collection takes place where local permits, processes, and policies are in place.

- To ensure interoperability with existing metadata standards, it is important to enter IPLC metadata into the appropriate fields consistent with both Dublin Core and Darwin Core. Providing robust provenance metadata for all IPLC samples and aTK, which includes place (geolocation), people (IPLC, researcher), process (sampling, permits, protocols), and date of collection information, helps to safeguard both fair attribution and benefit-sharing back to the partnering IPLC
- Respecting cultural sensitivities, customary laws, norms and protocols surrounding metadata is important when partnering with an IPLC, and in some cases redacting specific metadata fields may be warranted (e.g., the disclosure of culturally significant sites) or IPLCs may also wish for some metadata to be generalized (e.g., geographic coordinates).
- Retaining consistency amongst the metadata agreed upon to be collected and associated with IPLC genetics resources and aTK across the genomics data, vouchers, biobanked samples and publications helps to establish long term connections. between the genetics resources and its associated sequence information. This can help to prevent re-sampling and duplication of sequencing efforts.

Step 4 Ex-situ Samples: Taxonomy, Vouchering and Biobanking

- Consulting with IPLCs about storage options for ex-situ samples can help to provide a mutual understanding of the risks and benefits of ex-situ collection for both the project, the partnering IPLC and society at large.
- If ex-situ storage is agreed, prioritizing storage within an institution/organization/entity run by an IPLC partner or on the lands of the partnering IPLC is a highly encouraged standard of practice when feasible.
- Considerations for projects when managing ex-situ IPLC samples mutually agreed to be housed outside IPLC jurisdiction include:
    - Ensuring IPLC sample sharing and managing is in accordance with established consent and codified mutual agreements.
    - Providing standardized, documented procedures to support staff in stewarding IPLC samples.

- Retaining consistent metadata with that collected for the initial sample (see sample collection metadata recommendations) ensures all ex-situ samples can be linked to their associated genomics data.
- Prioritizing access requests of partnering IPLC(s), when possible.
- Recognizing IPLC species names as co-equal, and facilitating their coexistence with Western scientific names when valued by IPLC partners.

- If a project identifies an unethically or illegally collected IPLC sample, where possible it is encouraged to explore sample withdrawal and repatriation.

Step 5 Data

- Developing and agreeing upon a data management and sharing plan ensures responsible handling of data generated from species, or aTK, accessed within IPLC jurisdiction across the data lifecycle. Codifying this plan within a mutual agreement established prior to sample collection is highly encouraged for trust building, power-sharing and transparency.
- IPLCs will acquire greater benefits from project participation if long-term, sustained access to the data by the IPLC after project completion is prioritized.
- Aspects for projects to consider when developing a data management and sharing plan include:
    - Defining metadata collection procedures: Ensuring the agreed upon metadata is consistent and associated between the initial genetics resource, ex-situ samples, and sequence data ensures long term connections are established. See Sample Collection recommendations for culturally appropriate metadata and controlled vocabulary considerations.
    - Details on Access and Use: Defining access, use and re-use permissions, and any associated cultural protocols for utilization of the data that are respectful of Indigenous Data Sovereignty and uphold the wishes and rights of the partnering IPLC(s) is a crucial component if sharing IPLC data.

- Repository Description: Describing the digital repository of repositories that will manage and store the IPLC both temporarily (e.g., temporary cloud storage) and long term (e.g., Genbank).
- Outlining intended data analysis: Defining potential analysis to be conducted as part of the project, noting the importance of supporting meaningful research where outcomes benefit both the partnering IPLC and the research project.

● Directly involving IPLCs across all aspects of data handling can help to facilitate capacity building, knowledge transfer and act as a mode of non-monetary benefit-sharing. IPLCs may require a source of funding to engage in and take ownership of the data lifecycle themselves, in such cases projects could explore how best to financially support IPLC participation.

Step 6 Research Communication and Dissemination

● Co-designing a clear and meaningful research communication and dissemination plan can foster trust, ensure respectful communication of research outcomes, and act as a strategic tool to seek opportunities for securing future funding for project and partnership continuity.

● Considerations for projects when developing this plan include:

- Details on the objectives, proposed strategy and budget for communicating the research to external stakeholders and target audiences, prioritizing stakeholders identified as important to the IPLC.
- Defining the expectations and preferred method for IPLC evaluation of pre-published communications, including details on how IPLC feedback will be recognized prior to publication.
- Defining expectations for fair and equitable attribution of the IPLC in all communications and disseminations to each stakeholder.
- Establishing processes for resolution in cases where IPLC knowledge systems and feedback and research project outcomes are misaligned.

- Ensuring a mutual understanding exists in regards to the expectations of communication between the IPLC and the research project after the project has been completed.

Supplementary Information Section 2: Case Studies

Case Study 1.1. The Canadian North *Helicobacter pylori* Working Group

The CAN*Help* Working Group was established in 2006-2008 in partnership with Indigenous communities in Northern Canada, to address their concerns about a common stomach infection (*H. pylori*) that is a known risk factor for stomach cancer. The academic partnership was initiated by Aklavik, NWT community members who led the development of the community-driven approach, and supported by local health care providers and local and regional health decision-makers[1]. Each community that joined the partnership (with nine community projects initiating from 2007-2017) developed a local project planning committee, with membership and structures determined by local factors. The evolving Canadian policy[2] for research involving Indigenous data provided protections for communities who wished to be involved in advanced biomedical research but were concerned about the risks from such participation. This collaborative research program, which may not have existed without such strong Indigenous data protections, amassed a collection of *H. pylori* bacteria specimens isolated from 274 people, representing one of the only non-clinically derived collections of *H. pylori* specimens in the world. Community members have made the decision to share genomic data from some sequenced specimens[3], and work is underway to conduct full genome sequencing of the entire collection[4]. This advanced biological science was made possible through meaningful collaboration beginning with the identification of a research question to the ongoing governance of research outputs including genomic data and bacterial specimens.

Case Study 2.1. Maya ICBG Project

In 1998 a bioprospecting consortium, the International Cooperative Biodiversity Groups (ICBG), was awarded 2.5 million USD[5]. US stakeholders partnered with ECOSUR (a Mexican research and teaching center) and

Molecular Nature Ltd (a small Welsh biotechnology company). A fourth stakeholder that was not represented in the initial consortium was the Indigenous highland Peoples of Chiapas that span ~8,000 villages and speak four Mayan languages. The term community is heavily contested in Chiapas and there are a number of dynamic, sometimes conflicting, forms of territorial, economic, political and religious organizations. As a means to facilitate equitable benefit-sharing and to represent local interests, the project established a non–profit organization called PROMAYA that would receive ¼ of the project benefits of and be composed of a majority of Mayan representatives. From the outset and in line with CBD requirements of PIC and similar to the expectations that have since been laid down under the Nagoya Protocol, through national forums, information assemblies, flyers, radio spots and a one-act play performed in native languages to depict the information about the project. After this, 47 representatives were asked to sign a MOU expressing their interest in the project, 46 signed. In 1999, NGOs COMPITCH and RAFI launched a media campaign against the project, asserting the inadequacy of the consent process, arguing that important international patent and aTK debate information was not disclosed and that MOUs were not sufficiently signed as per customary law. The campaign also argued that both Maya speaking communities in Mexico and Guatemala should also have been asked for their PIC. The project was abandoned in 2001 due to disputes over who should have been consented and how. This case study highlights the importance of understanding the social, political and cultural structures that are in place prior to project initiation[6].

Case Study 2.2. Benefit-Sharing and Rooibos Tea

In 2019, after nine years of negotiation a rooibos industry wide benefit-sharing agreement was made with IPLCs in South Africa [7]. Here, the benefit-sharing agreement was based on aTK and implemented a levy of 1.5% on sales of unprocessed rooibos to be distributed equally back to the Khoi and San peoples[7]. As this product is already on the market it does not require translation time and so monetary benefits could be distributed immediately. Non-monetary benefits were also mentioned in the agreement.

Case Study 2.3. Intellectual Property and Basmati Rice

Most countries do not allow plant patents, but under US patent law new plant varieties can be protected. In the 1990s a Texan based company, RiceTec Inc[8], filed a patent on 'Basmati rice lines and grains' to the US Patent and Trademark Office. The patent caused outrage amongst Indian farmers and NGOs, in part due to the overly broad patent title. The patent itself only covered specific varieties of rice plants, the method for selective breeding, and propagation of particular rice grains. The Indian public, governmental, and international pushback spurred some patent claims to be retracted (claims 1-7,10 and 14-20). Other claims remain intact regardless of frustrations from other South Asian activists who claim the breeding techniques employed were not novel to the American inventors but are standard traditional practice. This was not acknowledged by the US Patent Office[9]. For additional examples see Annex Table 2.

Case Study 3.1. Bilberry Metadata Collection

The bilberry, *Vaccinium myrtillus L.*, a member of the Vacciniium genus along with cranberry and blueberry, are prized for their taste, nutraceutical, and health properties. Unlike the other members of this genus it has remained undomesticated, and it is almost entirely harvested from the wild. Bilberries can withstand exposure to the harsh winters of the Arctic Circle and have been an essential part of the diet and culture of Indigenous Peoples of Northern Europe, the Sámi. Since time immemorial bilberry's beneficial and disease-preventing nature has been recognised by the Sámi. Sámi reindeer herders utilized the berries together with reindeer milk and crowberries during harsh winter periods and to fight scurvy. New Zealand researchers produced the first chromosome-scale assembly for bilberry through accessing and utilizing samples obtained from Northern Europe[10]. In acknowledgement of the Sámi who have lived in this geographical area since time immemorial, and their aTK, the researchers placed a Biocultural Notice on the data with additional details outlined in the data availability statement. As this data sits within a public digital repository, disclosing a Notice provides an opportunity for future data users to also acknowledge the Sami' contribution to this dataset. Interestingly, bilberry has a cultural significance to many other communities across the world. Additional IPLC communities with aTK associated with the bilberry can also apply a Label or Notice to the data.

Case Study 3.1. Taxonomic Nomenclature Systems

Taxonomy is the pillar of biodiversity conservation responsible for the categorization and classification of species, but it has been plagued by its colonial legacy. The broadly adopted Linnaean system of identifying, naming, and classifying species proposed has erased IPLCs' names for fauna and flora and has frequently failed to recognize and credit their established knowledge of organisms. For instance, the native, culturally salient Aotearoa New Zealand taonga species, kauri and rewarewa, were renamed as *Agathis australis* and *Knightia excelsa* respectively. In the context of data-intensive science, the erasure of IPLC nomenclature appropriates their knowledge for use by Western researchers while perpetuating inequities of access and benefit from scientific research. Wehi et al. [12] recently piloted several approaches to build positive relationships between Maori names for birds and biodiversity research, including incorporating Maori names into environmental reports and interactive learning tools. Using scientific names to recognize and support Maori culture and aTK is also possible and discussed in Veale et al [13]. They highlight the importance of naming as deepening a relationship between people and nature, as pointed out by Manuka Hanare at the University of Auckland: "To name something is the means of establishing a relationship, namely a whakapapa, between the person or group doing the naming and the thing named. It is the basis upon which connections are made, identity clarified and asserted, and mana over that thing is generated" [13].

Case Study 3.2. Biobanking "For Natives, by Natives"

The Native BioData Consortium (NBDC) [14] was established in 2018 in Eagle Butte, South Dakota. It is the first not-for-profit institute and biobank that is housed within the sovereign borders of a tribal nation within the US. NBDC is led by Indigenous scientists and tribal community members. Indigenous Data Sovereignty is at the core of NBDC's mission, and is made possible through keeping samples and data local. Along with its role in providing long term, and sustained storage for Indigenous samples, its board members work on 1) building data science capacity both locally, and nationally through workshops (IndigiData [15]) and mentorship; 2) building trust with community members; and 3) developing policy, research projects and research practices that benefit local

Indigenous communities. NBDC enables long term, culturally aware, and sustainable storage for both samples and their associated metadata that have been obtained in partnership with Indigenous Peoples, including both samples from Peoples and the species found on Indigenous lands and waters. Its Indigenous governance structure allows for appropriate access, use, reuse and destruction of stored samples and associated metadata in a way that promotes research that is responsive to the needs of the communities that have provided those samples.

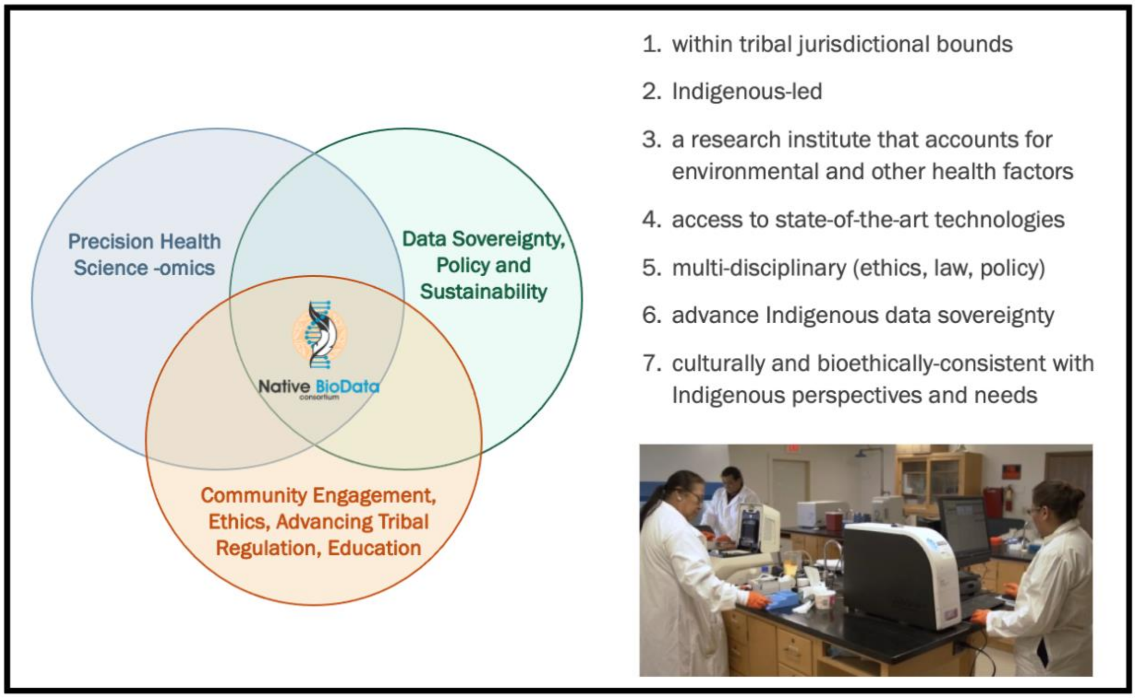

Supplementary Figure 2: The Native Biodata Consortium. Right: An overview of the unique nature and mission of the Native BioData Consortium. Left: Venn diagram highlighting the interdisciplinary breadth of the consortia.

Case Study 5.1. Genomics Aotearoa Data Repository

Genomics Aotearoa [16] is a national project to support culturally-aware genomics and bioinformatics research across Aotearoa, New Zealand. Māori are the Indigenous Peoples of Aotearoa, and Mātauranga Māori (aTK and values) is at the core of the project’s mission. For Māori, there are many species that are of significant cultural importance and are considered taonga (treasure), e.g. Kākāpō. In accordance with the Treaty of Waitangi [17], Māori are the rightful guardians of taonga, and in Māori tradition in order to retain this guardianship taonga must stay

within the shores of New Zealand. To ensure this right is respected, Genomics Aotearoa had the opportunity to build a genomics informatics infrastructure that was responsive to Māori needs. This infrastructure had to allow for the sustainable storage and Māori governance of Māori data. In line with this value, Genomics Aotearoa established the Genomics Aotearoa Data Repository [18]. The repository has come up with creative technical solutions to operationalize the FAIR and CARE principles, safeguarding Māori rights and values. The FAIR technical infrastructure allows Māori genomics and metadata to be found, in a format that is interoperable, accessible and reusable. The CARE-aligned procedures implemented by the repository support researchers in accessing and using data in a responsible and ethical manner while allowing Māori to tangibly benefit and govern Māori data through data access requests.

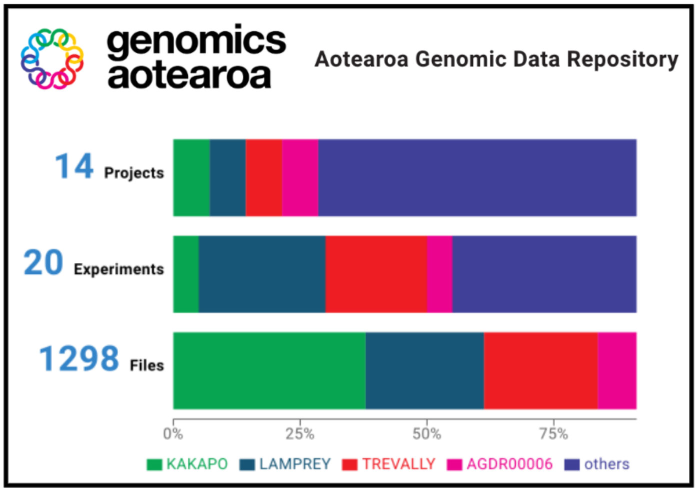

Supplementary Figure 3: Respectful and sustained storage of IPLC data. An illustration of the number of projects, experiments and files being stored in the Aotearoa Data Repository.

Case Study 6.1. A CONSERVCOM Project's Communication Plan

The mission of the project was to investigate the effectiveness of three types of forest management and conservation initiatives that used different strategies for local participation in decision making processes: protected areas, IPLC-conserved territories and areas, and payment exchange schemes [19]. IPLCs located in the states of Veracruz, Oaxaca, Campeche and Quintana Roo in Southeastern Mexico participated in the project.

From the outset, the project design included the development of a communication strategy that included plans for both academic (researchers, practitioners and other biodiversity conservation experts) and non-academic target audiences (local communities, NGOs, protected area managers and policymakers). The non-academic communication strategy was structured in three stages: 1) Understanding the communication context (Ethnography), 2) Fostering interaction and exchange to develop interactive communication tools (Dialogue/Focus groups with IPLCs), and 3) Workshops to validate the developed communication tools, correct misinterpreted/misrepresented findings from the research study, and add additional information that complemented research findings (Workshops)[19].

Supplementary Table 1: Proactive Dialogue Checklist for Researchers.

This checklist tool can be printed by researchers or research projects to guide a proactive dialogue with potential IPLC partners prior to project initiation. The outcomes of the discussion can then be used by the researcher/research project to assess the safety of the project and brainstorm ideas for plan development and project design improvement.

	Project Checklist	Why?	Topics to Discuss
☐	Objectives & Motivations	Clarity	- Project purpose, mission and goals, free from complex language<br>- Motivations and expectations of the IPLC
☐	Funding	Transparency	- Funding source, parameters and duration<br>- Budget for costs associated with partnership building
☐	Roles & Responsibilities	Accountability	- Define individual roles and responsibilities within the partnership
☐	Sample Handling & Governance Plan	Power-sharing	- Species proposal and motivations for collection<br>- Sample and metadata collection protocol<br>- Sample access, utilization, stewardship, and ex-situ storage<br>- Cultural sensitivities, permissions, protocols, or processes<br>- Sample return or destruction after sequencing
☐	Data Sharing & Management Plan	Power-sharing	- Types, and associated metadata<br>- Data storage<br>- Access, utilization, re-use, and sharing<br>- Downstream analysis<br>- Format accessibility for IPLC needs
☐	Benefit-sharing and Intellectual Property Plan	Transparency	- Potential risks for IPLC and society at large<br>- Potential Intellectual Property that may result from the project<br>- Potential monetary and non-monetary benefits that may result from the project
☐	Knowledge Transfer and Capacity Building Plan	Power-sharing	- Opportunities that benefit the IPLC (short- & long-term)<br>- Time and budget dedicated to executing plans

☐	Communication Plan	Power-sharing	- Internal communication strategy as project progresses<br>- External communication strategy to stakeholders<br>- Process of pre-approval prior to publications and fair attribution
☐	Completion	Sustainability	- Future collaboration potential- funding exploration<br>- Sustainable access to research outcomes, and data<br>- Long-term governance of samples/data outside of IPLC jurisdiction<br>-Documenting a partnership development protocol for future research use
	**Five Safes Framework Checklist**		**Project Improvement**
☐	Safe Data		How can we ensure that all data generated by the project is treated in a way that protects expressed IPLC concerns?
☐	Safe Project		How can we improve our project design to gain and maintain the approval of IPLC partners?
☐	Safe People		How can we train researchers and IPLC partners to ensure safe and authorized access to IPLC resources?
☐	Safe Settings		How can we create physical and digital environments that prevent unauthorized access and use of the provided IPLC resource (if housed outside of IPLC jurisdiction)?
☐	Safe Outputs		How can we share our research outputs to all project stakeholders safely?

Supplementary Table 2: Additional Resources. Resources for biodiversity genomics researchers participating in research in partnership with IPLCs.

Resource Name	Use	Ref
**IPLC Sovereignty**		
**Legislation**		
Convention on Biological Diversity		20
The Nagoya Protocol		21
United Nations Convention on the Law of the Sea		22
International Treaty on Plant Genetic Resources for Food and Agriculture		23

United Nations Declaration on the Rights of Indigenous Peoples		24
International Labor Organization (ILO) Convention No. 169		ref
UN International Covenant on Civil and Political Rights		25
UN International Covenant on Economic, Social and Cultural Rights		26
The International Convention on the Elimination of All Forms of Racial Discrimination calls		27
WTO Trade-Related Aspects of Intellectual Property Rights (TRIPS)		28
American Declaration on the rights of Indigenous Peoples		29
Wai 262 Aotearoa New Zealand		30
Treaty of Waitangi New Zealand		17
Nordic Saami Convention		31

Indigenous Peoples Rights Act 1997 Philippines		32
**Networks**		
United States Indigenous Data Sovereignty Network		33
Te Mana Raraunga, New Zealand Indigenous Data Sovereignty Network		34
Maiam nayri Wingara Aboriginal and Torres Strait Islander Data Sovereignty Collective		35
WHO PIP Framework		36
CITES		37
**Additional Sovereignty Resources**		
Indigenous Data Sovereignty - Toward an agenda	General Indigenous Competency	38
Indigenous Data Sovereignty and Policy	General Indigenous Competency	39

Indigenous Statistics	General Indigenous Competency	40
From Treaty Peoples to Treaty Nation		41
FAO Report on Forest governance by indigenous and tribal peoples	Report highlighting the important role IPLCs play in protecting and conserving Earth's biodiversity	42
The Role of Indigenous Peoples in Biodiversity Conservation: The Natural but Often Forgotten Partners	A report from the World Bank highlighting the importance of the contribution of IPLCs to conserving global biodiversity	43
**Section 1: Proactive Engagement & Partnership Building**		
UNESCO Guidelines for Engagement with Indigenous Peoples	Research Guidelines	44
Te Mata Ira Genome Research Guidelines	Research Guidelines	45
H3Africa Guidelines for Community Engagement	Research Guidelines	46
San Guidelines for Research	Research Guidelines	47
Framework for Research Engagement with First Nation, Metis, and Inuit Peoples	Engagement Framework	48
Guidelines for Ethical Research in Australian Indigenous Studies	Research Guidelines	49

FAO on Indigenous and Tribal Peoples	Guidelines	50
United Nations Resource Kit for Indigenous Issues		51
CARE Principles	Data Governance Framework	52
Decolonizing performances: deconstructing the global postcolonial in Handbook of Critical and Indigenous Methodologies	Research Framework	53
Decolonizing Methodologies: Research and Indigenous Peoples	Research Framework	54
US Tribal Research Codes	This paper includes references to a significant number of Tribal Research Codes from across the United States.	55
UNESCO Open Science Recommendations	Research Guidelines	56
OCAP	Data Governance Framework	57
A First Nations Data Governance Strategy	Data Governance Guidelines	58
Research Involving the First Nations, Inuit and Métis Peoples of Canada	General Guidelines for federally funded research. Note Chapter 9 specifically.	59
Ngā Tikanga Paihere: a framework guiding ethical and	An example of an Indigenous guideline that aligns with the 5 Safes Framework	60

culturally appropriate data use.		
Policy regarding research and project collaborations with Sámiid Riikkasearvi		61
Our Knowledge Our Way in Caring for Country		62
Code of Ethics of the International Society of Ethnobiology		63
**Section 2: Sample Collection and Processing**		
**Section 2.1: Access and Benefit Sharing**		
German Nagoya Protocol Hub	Contains extensive basic and advanced tools, and resources on the provisions of the Nagoya Protocol, researcher and institutional compliance, case studies and FAQs	64
CARE Principles	Data Governance Framework	52
United Nations FPIC: An Indigenous Peoples right and a good practice for Local Communities	UN FAO best practices document on FPIC with IPLCs	65
The Mo'otz kuxtal Voluntary Guidelines	Voluntary guidance on consent adopted by the CBD in pursuit of implementing Article 8j	66
Access and Benefit Sharing Clearing House	This website contains basic ABS procedures implemented by the Parties, IRCCs, and model contractual clauses	67
Nagoya Protocol Learning Portal	This portal contains useful basic and advanced information on the Nagoya Protocol and includes	68

	workshops, case-studies and guidelines.	
ABS BioTrade	Here you will find multimedia tools and publications that cover ABS projects, ABS background, Implementing the Nagoya Protocol, and ABS Cases	[69]
Toward Access and Benefit Sharing: Case Studies from the Pacific		[70]
Access and Benefit Sharing in Practice: Trends in partnerships across sectors		[71]
United Nations GEF Gender Responsive Approach Toolkit		[72]
**Section 2.2: Intellectual Property Rights**		
WIPO Traditional Knowledge Division	Extensive resources on Intellectual Property considerations specifically developed for IPLC samples, and associated Traditional Knowledge	[73]
WIPO Intellectual Property and Genetics Resources - Background Brief Number 10		[74]
WIPO A guide to Intellectual Property issues in Access and Benefit-Sharing Agreements		[75]
WIPO Key Questions on Patent Disclosure Requirements for Genetics Resources and Traditional Knowledge		[76]

WIPO Intellectual Property and Genetic Resources, Traditional Knowledge and Traditional Cultural Expressions		77
WIPO Protect and Promote your Culture: A Practical Guide to Intellectual Property for Indigenous Peoples and Local Communities		78
Patent landscape in biodiversity		79
WIPO Patent Disclosures		80
Examples of plant patents in Australia		81
**Section 2.3: Sample Metadata**		
CARE Principles	Data Governance Framework	52
FAIR Principles	Data Governance Framework	82
Local Context Hub	This hub has been developed to support the implementation of the Traditional Knowledge and Biocultural Labels. These labels can be associated with biosamples through metadata.	83
Earth BioGenome Project Metadata Collection Standards	This paper outlines the current minimum standards expected by the EBP for EBP reference genome production	84
European Reference Genome Atlas' Metadata Collection SOP and Manifest	This github repository contains ERGA's Standard Operating Procedures, and metadata collection manifest information	85

Section 3: Ex-situ Samples		
CARE Principles	Data Governance Framework	52
Local Context Hub	This hub has been developed to support the implementation of the Traditional Knowledge and Biocultural Labels. These labels can be associated to all ex-situ specimens housed in biobanks, and museum collections.	83
Rutzolijirisaxik Voluntary Guidelines	Voluntary guidance on repatriation adopted by the CBD in pursuit of implementing Article 8j	86
The critical importance of vouchers in genomics	A paper that outlines the importance of vouchering in genomics research	87
Cryoarks Resources	This website provides extensive documentation on the biobanking process including guidance, templates, training materials and request forms	88
Native Biodata Consortium	Example of a Native owned biorepository housed within Tribal jurisdiction	14
**Section 4: Data**		
CARE Principles	Data Governance Framework	52
TRUST Principles	Principles for Digital Repositories	89
FAIR Principles	Data Governance Framework	82
Global Indigenous Data Alliance	International network to promote Indigenous Data Sovereignty and Governance	90

Genomics Aotearoa Data Repository	Example of a Indigenous Data Repository	18
Local Context Hub	This hub has been developed to support the implementation of the Traditional Knowledge and Biocultural Labels. These labels can be associated with genomics data through metadata fields.	83
Rutzolijirisaxik Voluntary Guidelines	Voluntary guidance on repatriation adopted by the CBD in pursuit of implementing Article 8j	86
DSI Network	Biodiversity research community network tasked to discuss issues concerning DSI.	91
Earth BioGenome Project Statement on DSI	EBP position on the review of the scope of the Nagoya Protocol to include DSI	92
The United States Nagoya Protocol Action Group Statement on DSI	USNPAG on DSI and the Nagoya Protocol	93
CBD DSI Webpage	Resources issues by the Convention concerning the ongoing discussion to include DSI under the scope of the Nagoya Protocol	94
IndigiData	Example of an Capacity Building Training for Indigenous Peoples in Data Science	15
SING Consortium	Example of an Capacity Building Initiative for Indigenous Peoples in Genomics with workshops spanning Aotearoa, Australia, Canada, and United States	95
Tamamta Program	Example of an Capacity Building Training Program for Alaskan Natives	96

Section 5: Research Communication and Dissemination		
CARE Principles	Data Governance Framework	52
Local Context Hub and it's implementation in a research publication	This hub has been developed to support the implementation of the Traditional Knowledge and Biocultural Labels. Labels and notices can be attached to research publications e.g. bilberry genome assembly.	10,83
Molecular Ecology Resources	Data availability and Benefit-sharing statement	97
Nature Statement	Data availability information including samples	98

Supplementary Table 3: Interoperable Metadata. This table highlights the appropriate Rights metadata fields for Local Context Labels and Notices.

Field / attribute	Value	Example value(s)
**LABELS**		
Rights	The custom Label text created by a community for this collection/item using the Local Contexts Hub. For clarity, this should be preceded by the name of the Label. Do not include the generic URL for the Label.	Local Contexts TK Attribution: This Label is being used to correct historical mistakes or exclusions pertaining to this material. This is especially in relation to the names of the people involved in performing or making this work and/or correctly naming the community from which it originally derives. As a user you are being asked to also apply the correct attribution in any future use of this work.
rightsURI	The URL of the project in the Local Contexts hub.	https://localcontextshub.org/projects/29fc8acd-d647-48db-a766-b2c1bd9264b9/

rightsIdentifier	The identifier of the specific Label being applied.	TK-A, BC-P
rightsIdentifierScheme	Local Contexts	Local Contexts
schemeURI	Local Contexts website URI	https://localcontexts.org
**NOTICES**		
Rights	Notice text from the landing page of the Notice. For clarity, this should be preceded by the name of the Notice and the URL for the Notice. Use the exact text from the landing page; do not adapt or change the text.	Local Contexts Traditional Knowledge (TK) Notice https://localcontexts.org/notice/tk-notice: The TK Notice is a visible notification that there are accompanying cultural rights and responsibilities that need further attention for any future sharing and use of this material. The TK Notice may indicate that TK Labels are in development and their implementation is being negotiated.
rightsURI	The URL of the project in the Local Contexts hub.	https://localcontextshub.org/projects/29fc8acd-d647-48db-a766-b2c1bd9264b9/
rightsIdentifier	The identifier of the specific Notice being applied.	TK-Notice, BC-Notice, Attribution-Incomplete, Open-To-Collaborate
rightsIdentifierScheme	Local Contexts	Local Contexts

Supplementary References

1. Goodman, K. J. *et al.* Community-driven epidemiologic research: Guiding principles. *Global Epidemiology* **1**, 100013 (2019).
2. Government of Canada & Interagency Advisory Panel on Research Ethics. TCPS 2 (2018) – chapter 9: Research involving the first nations, Inuit and Métis peoples of Canada. https://ethics.gc.ca/eng/tcps2-eptc2_2018_chapter9-chapitre9.html (2019).

3. Kersulyte, D. *et al.* Complete Genome Sequences of Two Helicobacter pylori Strains from a Canadian Arctic Aboriginal Community. *Genome Announc.* **3**, (2015).

4. Funding decisions database - CIHR. https://webapps.cihr-irsc.gc.ca/decisions/p/project_details.html?applId=390832&lang=en (2008).

5. Berlin, B. *et al.* The Maya ICBG: drug discovery, medical ethnobiology, and alternative forms of economic development in the Highland Maya Region of Chiapas, Mexico. *Pharm. Biol.* **37**, 127 (1999).

6. Feinholz-Klip, D., García Barrios, L. & Cook Lucas, J. The Limitations of Good Intent: Problems of Representation and Informed Consent in the Maya ICBG Project in Chiapas, Mexico. in *Indigenous Peoples, Consent and Benefit Sharing: Lessons from the San-Hoodia Case* (eds. Wynberg, R., Schroeder, D. & Chennells, R.) 315–331 (Springer Netherlands, 2009).

7. Schroeder, D., Chennells, R., Louw, C., Snyders, L. & Hodges, T. The Rooibos Benefit Sharing Agreement–Breaking New Ground with Respect, Honesty, Fairness, and Care. *Camb. Q. Healthc. Ethics* **29**, 285–301 (2020).

8. RiceTec Inc. RiceTec. *RiceTec Inc* https://www.ricetec.com/.

9. Mukherjee, U. *A Study of the Basmati Case (India-US Basmati Rice Dispute): The Geographical Indication Perspective*. (SSRN, 2008).

10. Wu, C., Deng, C., Hilario, E. & Albert, N. W. A chromosome-scale assembly of the bilberry genome identifies a complex locus controlling berry anthocyanin composition. *Mol. Ecol.* (2022).

11. Wu, C. *et al.* A chromosome-scale assembly of the bilberry genome identifies a complex locus controlling berry anthocyanin composition. *Mol. Ecol. Resour.* **22**, 345–360 (2022).

12. Wehi, P. M. *et al.* Enhancing awareness and adoption of cultural values through use of Māori bird names in science communication and environmental reporting. *N. Z. J. Ecol.* **43**, 1–9 (2019).

13. Veale, A. J. *et al.* Using te reo Māori and ta re Moriori in taxonomy. *N. Z. J. Ecol.* **43**, 1–11 (2019).

14. Native BioData Consortium. Native BioData Consortium. *Native BioData Consortium* https://nativebio.org/.

15. IndigiData. IndigiData. *IndigiData* https://indigidata.nativebio.org/.

16. Aotearoa, G. Genomics Aotearoa. *Genomics Aotearoa* https://www.genomics-aotearoa.org.nz/.

17. *Te Tiriti o Waitangi / The Treaty of Waitangi, 1840*. (Department of Internal Affairs in association with Bridget Williams Books, 2017).

18. Aotearoa, G. Genomics Aotearoa Data Repository. *Genomics Aotearoa Data Repository* https://repo.data.nesi.org.nz/.

19. Ruiz-Mallén, I. Communicating Biodiversity Conservation Research Through Dialogue and Mutual Learning in Rural and Indigenous Communities. in *Biodiversity and Education for Sustainable Development* (eds. Castro, P., Azeiteiro, U. M., Bacelar-Nicolau, P., Leal Filho, W. & Azul, A. M.) 155–168 (Springer International Publishing, 2016).

20. United Nations Environment Programme. *Convention on Biological Diversity*. (Environmental Law and Institutions Programme Activity Centre, 1992).

21. United Nations Environmental Programme. Secretariat of the Convention on Biological Diversity. *Nagoya Protocol on Access to Genetic Resources and the Fair and Equitable Sharing of Benefits Arising from Their Utilization to the Convention on Biological Diversity: Text and Annex*. (Secretariat of the Convention on Biological Diversity, 2010).

22. Oxman. The regime of warships under the United Nations Convention on the Law of the Sea. *Va. J. Int'l L.*

23. Mekouar. A global instrument on agrobiodiversity: the International Treaty on Plant Genetic Resources for Food and Agriculture. *FAO legal papers online*.

24. Assembly, U. N. G. United Nations declaration on the rights of indigenous peoples. *UN*

Wash **12**, 1–18 (2007).

25. United Nations. International covenant on civil and political rights. Preprint at https://doi.org/10.18356/b2cd8230-en (2014).
26. United Nations. International covenant on economic, social and cultural rights. Preprint at https://doi.org/10.18356/a93bf03c-en (2014).
27. International Convention on the Elimination of All Forms of Racial Discrimination. *International Convention on the Elimination of All Forms of Racial Discrimination*. (United Nations Office of Public Information, 1983).
28. World Trade Organization. *The WTO Agreements: The Marrakesh Agreement Establishing the World Trade Organization and its Annexes*. (Cambridge University Press, 2017).
29. Law, I. American Declaration on the Rights of Indigenous Peoples. *Indian Law* (2017).
30. Geismar, H. Resisting settler-colonial property relations? The WAI 262 claim and report in Aotearoa New Zealand. *Settler Colonial Studies* **3**, 230–243 (2013).
31. Koivurova, T. The Draft Nordic Saami Convention: Nations Working Together. *Rev. Eur. Community Int. Environ. Law* **10**, 279–293 (2008).
32. Fitschen, T. Document. Protecting indigenous peoples and communities in the Philippines: the indigenous peoples rights act of 1997. *International Journal of Cultural Property* vol. 7 526–537 Preprint at https://doi.org/10.1017/s094073919877050x (1998).
33. United States Data Sovereignty Network. https://usindigenousdata.org/.
34. Te Mana Raraunga, New Zealand Indigenous Data Sovereignty Network. *Te Mana Raraunga* https://www.temanararaunga.maori.nz/ (2021).
35. nayri Wingara, M. Maiam nayri Wingara, Aboriginal and Torres Strait Islander Data Sovereignty Collective: About Us. Preprint at (2017).
36. *Pandemic Influenza Preparedness (PIP) Framework for the sharing of influenza viruses and access to vaccines and other benefits*. (World Health Organization, 2021).
37. *CITES, Text of the Convention on International Trade in Endangered Species of Wild Fauna*

and Flora: Signed March 3, 1973, Entered Into Force July 1, 1975. (U.S. Fish and Wildlife Service, Office of Management Authority, 1993).

38. Kukutai, T. & Taylor, J. *Indigenous data sovereignty: Toward an agenda*. (ANU Press, 2016).
39. Walter, M., Kukutai, T., Carroll, S. R. & Rodriguez-Lonebear, D. *Indigenous Data Sovereignty and Policy*. (Taylor & Francis, 2020).
40. Walter, M. & Andersen, C. *Indigenous statistics: A quantitative research methodology*. (Routledge, 2016). doi:10.4324/9781315426570.
41. Poelzer, G. & Coates, K. S. *From Treaty Peoples to Treaty Nation: A Road Map for All Canadians*. (UBC Press, 2015).
42. Food and Agriculture Organization of the United Nations & Fund for the Development of the Indigenous Peoples of Latina America and the Caribbean. *Forest governance by indigenous and tribal peoples: An opportunity for climate action in Latin America and the Caribbean*. (Food & Agriculture Org., 2021).
43. Sobrevila, C. *The role of indigenous peoples in biodiversity conservation: The natural but often forgotten partners*. (2008).
44. UNESCO. *UNESCO policy on engaging with indigenous peoples*. https://unesdoc.unesco.org/ark:/48223/pf0000262748 (2018).
45. Hudson, M. *et al.* Te Mata Ira: Guidelines for Genomic Research with Māori.: Te Mata Ira: Guidelines for Genomic Research with Māori. (2016).
46. H3Africa Working Group on Ethics and Regulatory Issues for the Human Heredity and Health in Africa. *H3Africa Consortium*. vol. 3 (H3Africa, 2017).
47. Callaway. San people issue research code. *Nature*.
48. University of Manitoba. Framework for research engagement with first nation, Metis, and Inuit peoples. Preprint at (2013).
49. Australian Institute of Aboriginal and Torres Strait Islander Studies. *Guidelines for Ethical*

Research in Australian Indigenous Studies. https://ich.unesco.org/doc/src/Example-code_of_ethics-Australian_Institute_of_Aboriginal_and_Torres_Strait_Islander_Studies.pdf (2012).

50. Food and Agriculture Organization of the United Nations. *FAO Policy on Indigenous and Tribal Peoples*. http://www.fao.org/3/i1857e/i1857e00.pdf.
51. Nations, U. Resource Kit on Indigenous Peoples' Issues. Preprint at (2008).
52. CARE Principles of indigenous data governance — global indigenous data alliance. https://www.gida-global.org/care.
53. Blue & Mutua. Decolonising Performances: Deconstructing the Global Postcolonial. *Handbook of Critical and Indigenous Methodologies*.
54. Smith, L. T. *Decolonizing Methodologies: Research and Indigenous Peoples*. (Zed Books Ltd., 2013).
55. Carroll, S. R. *et al.* Using Indigenous Standards to Implement the CARE Principles: Setting Expectations through Tribal Research Codes. *Front. Genet.* **13**, 823309 (2022).
56. Hampson, DeSart & Steinhauer. Open Science Roadmap: Recommendations to UNESCO. *Open Sch.. Initiat. Proc.*
57. First Nations Information Governance Centre. The First Nations Principles of OCAP®. *The First Nations Principles of OCAP®* https://fnigc.ca/ocap-training/.
58. First Nations Information Governance Centre. *A First Nations Data Governance Strategy*. (2020).
59. Canadian Institutes of Health Research, N. S., Engineering Research Council of Canada, Sciences, S. & of Canada, H. R. C. Tri-Council Policy Statement: ethical conduct for research involving humans--TCPS 2. Preprint at (2018).
60. Stats, N. Z. Ngā Tikanga Paihere: a framework guiding ethical and culturally appropriate data use. https://www.data.govt.nz/assets/data-ethics/Nga-Tikanga/Nga-Tikanga-Paihere-Guidelines-December-2020.pdf (2020).

61. Riikkasearvi, S. Policy Regarding Research and Project Collaborations with Sámiid Riikkasearvi. Preprint at (2019).

62. Woodward, E., Hill, R., Harkness, P. & Archer, R. Our Knowledge Our Way in caring for Country: Indigenous-led approaches to strengthening and sharing our knowledge for land and sea management. Best Practice Guidelines from Australian Experiences. (2020).

63. of Ethnobiology, I. S. ISE Code of Ethics (with 2008 additions). Preprint at (2006).

64. Federal Agency for Nature Conservation. German Nagoya Protocol Hub. *German Nagoya Protocol Hub* https://www.nagoyaprotocol-hub.de/.

65. Food and Agriculture Organization of the United Nations *et al. Free Prior and Informed Consent: An Indigenous Peoples' Right and a Good Practice for Local Communities : Manual for Project Practitioners*. (Food and Agriculture Organization of the United Nations, 2016).

66. Parks, L., Peters, W. & Lennan, M. Guidelines and Codes on the Participation of Indigenous Peoples and Local Communities of the Convention on Biological Diversity: A Comparative Analysis Using Natural Language Processing. (2019) doi:10.2139/ssrn.3384691.

67. Diversity, C. on B. Access and Benefit-sharing Clearing House. *ABS Clearing House* https://absch.cbd.int/en/.

68. USA Nagoya Protocol Action Group. Nagoya Protocol Learning Portal. *Nagoya Protocol Learning Portal* https://learnnagoya.com/.

69. Biotrade, A. B. S. Access and Benefit-sharing BioTrade. *ABS Biotrade* https://www.abs-biotrade.info/.

70. Robinson. Towards access and benefit-sharing best practice: Pacific case studies. *DSEWPAC, Canberra and GIZ, Eschborn*.

71. Laird, S. A. & Wynberg, R. Access and benefit-sharing in practice: trends in partnerships across sectors. in (Secretariat of the Convention on Biological Diversity, 2008).

72. UN-GEF Global ABS Project. *Mainstreaming Gender in to ABS value chains: Gender*

Toolkit. (2019).

73. World Intellectual Property Organisation. WIPO Traditional Knowledge Division. *World Intellectual Property Organisation* https://www.wipo.int/about-wipo/en/activities_by_unit/index.jsp?id=122.

74. World Intellectual Property Organization (WIPO). *Intellectual property and genetic resources*. (Unknown).

75. Oliva, M. J., Rukundo, O. & World Intellectual Property Organization. *A Guide to Intellectual Property Issues in Access and Benefit-sharing Agreements*. (WIPO, 2018).

76. World Intellectual Property Organization. *Key questions on patent disclosure requirements for genetic resources and traditional knowledge*. (Unknown).

77. World Intellectual Property Organization,. *Intellectual property and genetic resources, traditional knowledge and traditional cultural expressions*. (Unknown).

78. World Intellectual Property Organization (WIPO). *Protect and promote your culture*. (Unknown).

79. Simmonds, M. S. J. *et al.* Biodiversity and patents: Overview of plants and fungi covered by patents. *Plants People Planet* **2**, 546–556 (2020).

80. Genetic resources. https://www.wipo.int/tk/en/genetic/.

81. Janke. From smokebush to spinifex: Towards recognition of Indigenous knowledge in the commercialisation of plants. *International Journal of Regional, Rural and Remote* doi:10.3316/informit.560547065367151.

82. Wilkinson, M. D. *et al.* The FAIR Guiding Principles for scientific data management and stewardship. *Scientific Data* **3**, 160018 (2016).

83. Contexts, L. Local Context Hub. *Local Contexts* https://localcontexts.org/.

84. Lawniczak, M. K. N. *et al.* Standards recommendations for the Earth BioGenome Project. *Proc. Natl. Acad. Sci. U. S. A.* **119**, (2022).

85. European Reference Genome Atlas Metadata. European Reference Genome Atlas Metadata

Collection Process. Preprint at https://github.com/ERGA-consortium/ERGA-sample-manifest (2022).

86. Secretariat of the Convention on Biological Diversity. *Rutzolijirisaxik Voluntary Guidelines for the Repatriation of Traditional Knowledge of Indigenous Peoples and Local Communities Relevant for the Conservation and Sustainable Use of Biological Diversity*. (2019).
87. Buckner, Sanders, Faircloth & Chakrabarty. Science Forum: the critical importance of vouchers in genomics. *Elife*.
88. CryoArks. CryoArks Resources. *CryoArks* https://www.cryoarks.org/resources/.
89. Lin, D. *et al.* The TRUST Principles for digital repositories. *Sci Data* **7**, 144 (2020).
90. Global Indigenous Data Alliance. https://www.gida-global.org/.
91. DSI Network. Digital Sequence Information Network. *DSI Network* https://www.dsiscientificnetwork.org/.
92. Earth Biogenome Project. *The Convention on Biological Diversity, the Nagoya Protocol, and Digital Sequence Information: Principles of the Earth BioGenome Project*. (2022).
93. The United States Nagoya Protocol Action Group. (2020).
94. Diversity, C. on B. Convention on Biological Diversity Webpage on Digital Sequence Information. *Convention on Biological Diversity Website* https://www.cbd.int/dsi-gr/.
95. SING Consortium. Summer Internship for Indigenous Peoples in Genomics Consortium. *SING Consortium* https://www.singconsortium.org/.
96. Tamamta. Tamamta Program. *Tamamta* https://www.tamamta.org/.
97. Wiley Online Library. *Author Guidelines*. https://onlinelibrary.wiley.com/page/journal/1365294x/homepage/forauthors.html (2019).
98. Nature, S. Data Availability Statement. *Springer Nature* https://www.springernature.com/gp/authors/research-data-policy/data-availability-statements/12330880.
